# 

**Published:** 1902-05

**Authors:** 


					﻿eza
-«r
a_
LU
cc
O-
LU
CS
C/9
a
o_
1
o
<
Ld
O
ID
CM
40
O
2
o"
o
0)
0)"
0
I
o-
0)
I
Is-
0)
I
(0
0)
co
U_
CO
C/9
LU
O-
a
C_3
CZJ
CD
fiQ
LEADS VETERINARY JOURNALISM IN AMERICA.
Vol* XXIII.
MAY, 1902.
No. 5
PUBLISHED MONTHLY AT $3 A YEAR. SINGLE COPIES, 30 CENTS.' ,
FOREIGN SUBSCRIPTIONS J3.50 PER YEAR.
THE JOURNAL
OF
Comparative Medicine
AND
VETERINARY ARCHIVES
EDITED AND PUBLISHED BY
W. HORACE HOSKINS, D.V.S.
00
o
c
z
□
o
o
2
m
(D
O
w
<0
o
V
D
m
00
Fl
>
□
<
"H
O
00
□
Fl
r
<
Fl
<
ALL COMMUNICATIONS AND PAYMENTS SHOULD BE ADDRESSED TO
Or. W. Horace Hoskins, Managing editor, 3452 Ludlow St., Philadelphia, Pa.
Copies may be had on order of all news-dealers at home and abroad.
AN ADVERTISING MEDIUM UNEQUALLED IN THE VETERINARY FIELD
				

